# Molecular and Serological Surveillance for *Mycobacterium leprae* and *Mycobacterium lepromatosis* in Wild Red Squirrels (*Sciurus vulgaris*) from Scotland and Northern England

**DOI:** 10.3390/ani14132005

**Published:** 2024-07-07

**Authors:** Zijie Zhou, Anouk van Hooij, Gaby N. Wassenaar, Emma Seed, Els M. Verhard-Seymonsbergen, Paul L. A. M. Corstjens, Anna L. Meredith, Liam A. Wilson, Elspeth M. Milne, Katie M. Beckmann, Annemieke Geluk

**Affiliations:** 1 Department of Infectious Diseases, Leiden University Medical Center, 2333 ZA Leiden, The Netherlands; z.zhou@lumc.nl (Z.Z.); a.van_hooij@lumc.nl (A.v.H.); g.n.wassenaar@lumc.nl (G.N.W.); e.m.verhard@lumc.nl (E.M.V.-S.); 2 The Royal (Dick) School of Veterinary Studies, The Roslin Institute, University of Edinburgh, Edinburgh EH25 9RG, UK; e.m.seed@sms.ed.ac.uk (E.S.); liam.wilson@ed.ac.uk (L.A.W.); elspeth.milne@ed.ac.uk (E.M.M.); katie.beckmann@ed.ac.uk (K.M.B.); 3 Department of Cell and Chemical Biology, Leiden University Medical Center, 2333 ZA Leiden, The Netherlands; p.l.a.m.corstjens@lumc.nl; 4 Faculty of Natural Sciences, Keele University, Keele ST5 5BG, UK; a.l.meredith@keele.ac.uk

**Keywords:** anti-PGL-I antibody, DNA, leprosy, *Mycobacterium leprae*, *Mycobacterium lepromatosis*, red squirrel, real-time PCR, UCP-LFA

## Abstract

**Simple Summary:**

Currently, the Eurasian red squirrel (*Sciurus vulgaris*) is the only known wild rodent detected with *Mycobacterium leprae* and *M. lepromatosis* infection and serves as a potential maintenance host for these mycobacteria. Studies have detected leprosy bacilli in red squirrels from around the British Isles. However, red squirrels on the European mainland have not been shown to be infected with leprosy bacillus. To gain further insight into presence of leprosy bacilli in red squirrels in the northern UK, this study utilized samples from opportunistically collected red squirrel carcasses found since 2004. To specifically detect the presence of *M. leprae* and *M. lepromatosis* DNA, squirrel pinna was assayed by real-time PCR (qPCR). Additionally, we investigated whether specific antibodies directed against leprosy bacilli were present in blood/body cavity fluid from these and other animals. The rate of detection of either of these bacilli in the sampled red squirrels was 22.6%, indicating that leprosy bacilli remain present in the northern UK red squirrel population. Therefore, continuous monitoring for the presence of leprosy bacilli in red squirrels, using macroscopic examination alongside both molecular and serological assays, as well as further research to elucidate the ecology of the disease, is recommended.

**Abstract:**

Leprosy is a poverty-associated infectious disease in humans caused by *Mycobacterium leprae* or *M. lepromatosis*, often resulting in skin and peripheral nerve damage, which remains a significant public health concern in isolated areas of low- and middle-income countries. Previous studies reported leprosy in red squirrels in the British Isles, despite the fact that autochthonous human cases have been absent for centuries in this region. To investigate the extent of *M. leprae* and *M. lepromatosis* presence in wild red squirrels in the northern UK, we analyzed 220 blood/body cavity fluid samples from opportunistically sampled red squirrels (2004–2023) for specific antibodies against phenolic glycolipid-I, a cell wall component specific for these leprosy bacilli. Additionally, we assessed bacillus-derived DNA by real-time PCR (qPCR) in 250 pinnae from the same cohort. *M. lepromatosis* and *M. leprae* DNA were detected by qPCR in 20.4% and 0.8% of the squirrels, respectively. No cases of co-detection were observed. Detectable levels of anti-PGL-I antibodies by UCP-LFA were observed in 52.9% of animals with the presence of *M. lepromatosis* determined by qPCR, and overall in 15.5% of all animals. In total, 22.6% (*n* = 296) of this UK cohort had at least some exposure to leprosy bacilli. Our study shows that leprosy bacilli persist in red squirrels in the northern UK, emphasizing the necessity for ongoing molecular and serological monitoring to study leprosy ecology in red squirrels, gain insight into potential zoonotic transmission, and to determine whether the disease has a conservation impact on this endangered species.

## 1. Introduction

Leprosy, caused by infection with *Mycobacterium leprae* or *Mycobacterium lepromatosis*, is characterized by skin lesions and peripheral nerve damage and presents with a spectrum of clinical symptoms in humans. Due to the impact of the COVID-19 pandemic, there was a surge in new reported cases in humans globally in 2021, with a total of 140,594 cases, an increase of 10.2% compared to 2020. These numbers highlight the continued need for substantial investments in disease control [1].

While the majority of global human leprosy cases are attributed to *M. leprae* infection, *M. lepromatosis* was identified as the second causative mycobacterium in 2008 [2]. To date, *M. lepromatosis* cases have been reported from 11 countries in the Americas and Asia [3,4]. Due to the inability to cultivate leprosy bacilli in vitro [5,6], detection of the presence and identification of bacteria typically involves polymerase chain reaction (PCR) to amplify multicopy sequences of *M. leprae* (RLEP) [7] and *M. lepromatosis* (RLEM) [8] in the genome or specific genomic regions (contig 202 of *M. lepromatosis*) [9].

Leprosy can be classified according to the Ridley Jopling Classification [10], ranging from tuberculoid leprosy (TT), characterized by low bacterial load and primarily an elicitation of Th1 and Th17 T-cell immune responses [11,12,13], to lepromatous leprosy (LL), which features a high bacterial load and typically manifests as severe, disseminated infection with an anti-inflammatory Th2 response [14]. Additionally, patients infected with *M. lepromatosis* may exhibit a rare and more severe form known as diffuse lepromatous leprosy (DLL) [2,15]. According to the WHO classification, multibacillary leprosy (MB) refers to cases with more than five skin lesions [16], which typically display detectable antibody levels, particularly IgM targeting phenolic glycolipid-I (PGL-I) [17,18], a cell wall component of both *M. leprae* and *M. lepromatosis* [19]. Anti-PGL-I IgM levels correlate with an individual’s bacterial load [20,21,22], making it a useful biomarker for detecting infections caused by both leprosy bacilli [19]. To quantify anti-PGL-I IgM levels in a field-friendly manner, we have previously developed a lateral flow assay based on up-converting phosphor particles (UCP-LFA) [21,23,24]. This UCP-LFA has been successfully applied to armadillos [25] and red squirrels [26,27].

The population of Eurasian red squirrels (*Sciurus vulgaris*) in the UK has declined substantially over the last 70 years, due to threats including interspecific competition from gray squirrels (*Sciurus carolinesis*) introduced in the late 1800s [28]; squirrelpox disease due to infection with parapoxvirus (squirrel poxvirus), which is transmitted from and carried asymptomatically by gray squirrels; and habitat loss and fragmentation [29,30,31,32]. Currently, the red squirrel is protected as an endangered species under UK law and the Bern Convention [33]. The remaining UK populations of red squirrels are mostly in Scotland and northern England (northern UK) [34,35] with isolated populations also found on the Isle of Wight, Brownsea Island [36], the islands of Anglesey in Wales [37], and Jersey [38].

Leprosy bacilli were first identified in red squirrels in Scotland in 2014 [39]. The Eurasian red squirrel is the only wild rodent in which infection with leprosy bacilli has been detected in populations in the UK. Most infections in red squirrels in the UK have been identified as *M. lepromatosis*, whilst *M. leprae* has been found in smaller numbers of squirrels on the isle of Arran (Scotland) and Brownsea Island in recent years [40,41]. Squirrels with overt clinical leprosy typically present with skin lesions on their pinnae (external ears), eyelids, muzzle, and limbs [42]. However, *M. lepromatosis* has also been detected in clinically unaffected red squirrels, which has been interpreted as asymptomatic infection [41], possibly reflecting a state also observed in humans.

Despite human leprosy cases having been absent for centuries in the UK [43], it is clear that squirrels remain hosts for *M. leprae* and *M. lepromatosis* in the present day [44]: their role in the ecology of the disease remains to be elucidated. Monitoring leprosy bacilli in red squirrels is crucial for identifying potential zoonotic risks and understanding any population-level impact in this endangered species. To gain more insight into the extent to which leprosy bacilli have been present in the UK red squirrel population, we analyzed samples from red squirrels opportunistically received for post-mortem examination as part of a disease surveillance project at the Royal (Dick) School of Veterinary Studies (R(D)SVS), University of Edinburgh. The carcasses had been submitted over the past two decades (2004–2023). This analysis used molecular (qPCR) and immunological (UCP-LFA) methods to directly and indirectly investigate the presence of these mycobacteria.

## 2. Materials and Methods

### 2.1. Squirrel Samples

Eurasian red squirrel carcasses (*n* = 297; 150 male, 139 female, and 8 unknown) had been found in Scotland (*n* = 293) and northern England (*n* = 4) between 2004 and 2023. They were categorized by age (deduced through body weight and reproductive activity) [30] as 18 juveniles (<16 weeks), 41 subadults, 232 adults, and 6 of unknown age (Table S1). In 134 cases, trauma (such as roadkill) was the confirmed or probable cause of death; 116 cases were considered to have died of disease by post-mortem examination [45], such as squirrelpox, pneumonia, or enteritis, and the cause of death was unknown in 47 cases. Most of the squirrels had no gross post-mortem findings consistent with leprosy. One pinna sample per animal (*n* = 251) had been collected at the time of post-mortem examination and stored in approximately 1 mL 70% ethanol at refrigeration temperature (3 °C to 5 °C) before being analyzed. Blood had been preferentially sampled from each case, although this was not always possible due to various factors such as extensive external hemorrhage and post-mortem decay. In such circumstances, body cavity (thoracic and/or abdominal) fluid had been collected as an alternative where possible. These blood or body cavity fluid samples had been stored frozen at −20 °C. For analysis, these blood and body cavity fluid samples (*n* = 220) were defrosted, centrifuged, and the supernatants collected and preserved in tubes (Eppendorf, Hamburg, Germany) at −20 °C until further use.

### 2.2. DNA Extraction

DNA was isolated using the QIAamp UCP Pathogen Mini Kit (Qiagen, Germantown, MD, USA), as per the manufacturer’s instructions with modifications. In brief, pinnae were repeatedly serially sectioned with sterile surgical blades and transferred to microtubes (Sarstedt, Nümbrecht, Germany) containing 400 µL ATL buffer (provided in the kit) and three sterile 3 mm soda-lime glass beads (Avantor, Radnor, PA, USA). Tubes were then placed into a tissue homogenizer (Precellys 24, Bertin, Montigny-le-Bretonneux, FRANCE) applying two repeats of maximum velocity, each of 45 s duration. Next, 20 µL proteinase K (provided in the QIAamp kit) was added and the tubes were incubated in a thermomixer at 55 °C and 1300 rpm for two hours. After centrifugation at 10,000× *g* for 1 min, the supernatant was transferred to new microtubes containing 250 µL of 0.1 mm zirconia beads (Biospec, Bartlesville, OK, USA) and homogenized by two repeats of maximum velocity for 45 s, as before. Following centrifugation at 10,000× *g* for 1 min, the supernatants were transferred to new empty microtubes. Proteinase K (20 μL) was added, and the tubes were incubated in a thermomixer at 55 °C and 1300 rpm for 30 min. Next, 200 µL APL2 buffer was added, and tubes were incubated in a thermomixer at 70 °C and 1300 rpm for 10 min. Column extraction was performed after absolute ethanol precipitation (300 µL), as per the manufacturer’s instructions.

### 2.3. Real-Time PCR

Detection of *M. leprae* and *M. lepromatosis* was performed by a multiplex qPCR including *M. leprae* repetitive element (RLEP, 129 bp, including at least 28 copies) [7] and 168 bp fragment in contig 202 (including two copies) of *M. lepromatosis*, as previously described [9]. In brief, the amplification was performed in a final volume of 25 µL by the addition of 12.5 μL GoTaq™ Probe qPCR Master Mix (Promega, Madison, WI, USA), 0.9 μM forward and reverse primers, 0.25 μM TaqMan probes, and 5 μL template DNA. Nuclease-free water was used as for negative controls and either *M. leprae* Thai-53 (1.25 ng per qPCR reaction) or *M. lepromatosis* DNA (0.115 ng per qPCR reaction) for positive controls. DNA was amplified using the following profile: 2 min at 95 °C followed by 40 cycles of 15 s at 95 °C and 1 min at 60 °C on a QuantStudio 6 Flex Real-Time PCR System (Applied Biosystems, San Francisco, CA, USA). Samples were considered positive for the presence of *M. leprae* or *M. lepromatosis* DNA if the RLEP or 202 qPCR resulted in a cycle threshold (Ct) lower than 30. Samples with Ct values ≥ 35 were considered negative for the presence of bacterial DNA, while samples with Ct values between 30 and 35 were considered indeterminate.

### 2.4. Anti-PGL-I UCP-LFA

LFAs were performed with luminescent up-converting reporter particles (UCP). UCP nanomaterials (200 nm NaYF_4_:Yb^3+^,Er^3+^ particles, functionalized with carboxyl groups) were obtained from Intelligent Material Solutions Inc. (Princeton, NJ, USA). UCP conjugates were prepared with goat anti-human IgM (I0759, Sigma-Aldrich, St. Louis, MO, USA) at a concentration of 50 μg antibody per mg UCP according to the method described previously [46].

The LF strips (4 mm width) for detection of anti-PGL-I IgM were produced as described previously [23,24,47,48,49]. The test (T) line comprised 100 ng synthetic PGL-I (natural phenolic trisaccharide functionalized with a hexanoic acid linker for conjugation to BSA, NTP1-H-BSA and the flow control (FC) line comprised 100 ng rabbit anti-goat IgG (G4018, Sigma-Aldrich, St. Louis, MO, USA). UCP reporter conjugate (400 ng) was dried into the sample/conjugate release pad using a buffer containing 5% (*w*/*v*) sucrose. LF strips were stored at ambient temperature in plastic containers with a silica dry pad.

Supernatant was collected after centrifuging the blood/body cavity fluid, and 50 μL was diluted 1:50 (anti-PGL-I IgM) in assay buffer (100 mM Tris pH 8, 270 mM NaCl, 1% (*w*/*v*) BSA, 1% (*v*/*v*) Triton X-100). Diluted samples (50 μL) were applied to LF strips. Upon completion of LF, strips were analyzed with a UCP-dedicated benchtop reader (UPCON; Labrox, Turku, Finland). Results were displayed as the ratio value (R) of T and FC signals (peak area) based on relative fluorescence units (RFUs).

### 2.5. Anti-PGL-I Antibody ELISA

The levels of anti-PGL-I IgM in red squirrels’ sera were measured by ELISA as described previously with minor adjustments [25]; in brief, 96-well PolySorp NUNC plates (Thermo Fisher Scientific, Rochester, NY, USA) were coated with 50 μL of the NTP1-H-BSA (4 μg/mL) in 100 mM Na_2_CO_3_/NaHCO_3_ buffer (pH 9.6) at 4 °C overnight. Coated plates were blocked with 200 μL PBS/1% BSA/0.05% Tween 80 per well for 1 h, and 50 μL of 1:20 diluted sample was added and incubated for 2 h at room temperature. Then, 50 μL of goat anti-mouse IgM-HRP (1:10,000, A97230, Abcam, Cambridge, UK) or anti-human IgM-HRP (1:8000, A6907, Sigma-Aldrich, St. Louis, MO, USA) in 0.05% Tween 20/PBS was incubated for 2 h. In between each step, the wells were washed 3 times with PBS/0.05% Tween 20. A total of 50 μL of 3,3′,5,5′-Tetramethylbenzidine (TMB, Thermo Fisher Scientific, Rochester, NY, USA) was added, and the color reaction was stopped using H_2_SO_4_ after 10 min. Absorbance was determined at a wavelength of 450 nm (SpectraMax i3x, Molecular Devices, San Jose, CA, USA). The optical density at 450 nm (OD_450_) of the samples was corrected with the background OD (0.1% BSA in coating buffer).

### 2.6. M. leprae Genotyping

To determine the single nucleotide polymorphism (SNP) genotype of *M. leprae* (1, 2, 3 or 4), SNP-14676 (locus 1), SNP-1642875 (locus 2), and SNP-2935685 (locus 3) PCRs followed by Sanger sequencing were performed as described previously [20,50,51]. In brief, PCRs were performed using a PCR mixture containing 10 μL 5x Gotaq^®^ Flexi buffer (Promega, Madison, WI, USA), 5 μL MgCl_2_ (25 mM), 2 μL dNTP mix (5 mM), 5 μL forward and reverse primers (2 μM), 0.25 μL Gotaq^®^ G2 Flexi DNA Polymerase (5 u/μL), and 5 μL template DNA in a final volume of 50 μL. DNA was denatured for 2 min at 95 °C; followed by 45 cycles of 30 s at 95 °C; 30 s at 50 °C (locus 1), 55 °C (locus 2), or 53 °C (locus 3); 30 s at 72 °C; and a final extension cycle of 10 min at 72 °C on a Veriti™ 96-Well Fast Thermal Cycler (Thermo Fisher Scientific, Rochester, NY, USA). PCR products showing a band were purified prior to sequencing using the Wizard SV Gel and PCR Clean-Up System (Promega, Madison, WI, USA). Sequencing was performed on the ABI3730xl system (Applied Biosystems, Foster City, CA, USA) using the BigDye Terminator Cycle Sequencing Kit (Thermo Fisher Scientific, Waltham, MA, USA). Sequences were analyzed using Bioedit v7.0.5.3.

### 2.7. Statistical Analysis

Graphpad Prism version 10.0 for Windows (GraphPad Software, San Diego CA, USA) was used to perform Mann–Whitney U tests, Kruskal–Wallis with Dunn’s correction for multiple testing, and Fisher’s exact test and to compute Spearman correlation coefficients. The statistical significance level used was *p* ≤ 0.05.

## 3. Results

### 3.1. Detection of M. leprae and M. lepromatosis DNA in Red Squirrels

A total of 251 pinna and 220 blood/body cavity fluid samples collected from 297 squirrel carcasses from 2004 to 2023 (blood/body cavity fluid) or 2014 to 2023 (pinna), were analyzed (Table S1). For one animal, DNA isolation failed due to the limited sample amount.

**Figure 1:**
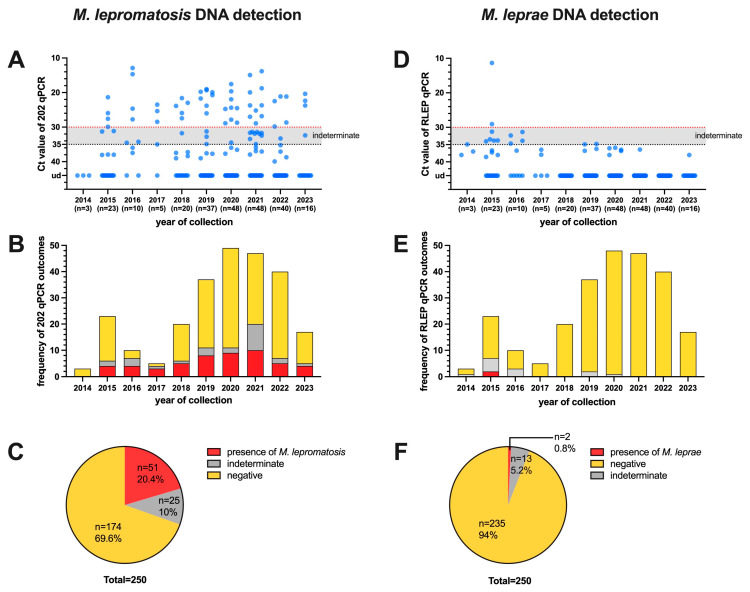
Presence of *M. lepromatosis* and *M. leprae* in red squirrels found between 2014 and 2023 by qPCR. The DNA samples (*n* = 250) derived from the pinna of red squirrels were assessed for the presence of 202 (left panel; **A**–**C**) and RLEP (right panel; **D**–**F**) sequences by qPCR. Ct values ≤ 30 were considered to indicate the presence of mycobacteria, Ct values ≥ 35 were considered negative, and 30 < Ct values < 35 were considered indeterminate for leprosy bacilli DNA. The Ct values for each animal (**A**,**D**) and frequency of outcomes (**B**,**E**) are represented on the *y*-axis, and the years of collection represented on the *x*-axis. The proportion for each of the three outcomes are provided in (**C**,**F**). ud, undetermined.

**Figure 2:**
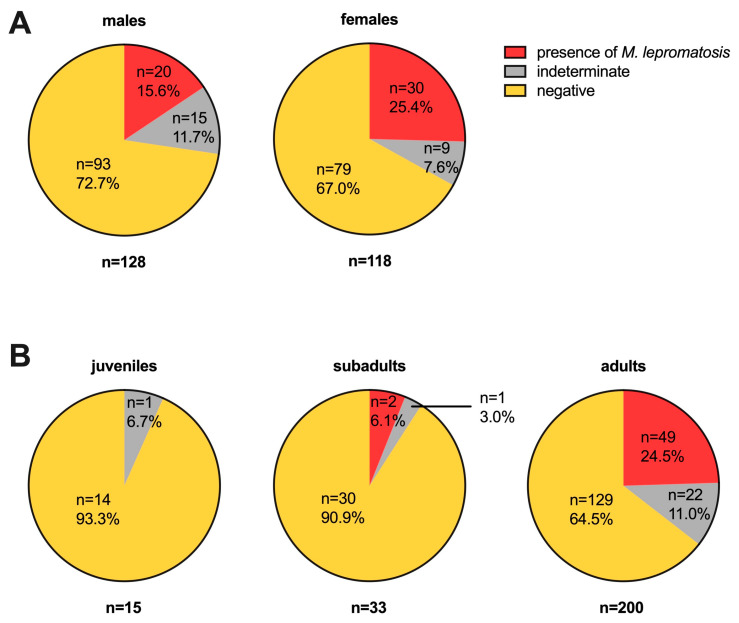
Categorization of *M. lepromatosis* presence. The outcomes of 202 qPCR in red squirrels found between 2014 and 2023 are summarized by sex (**A panel**: 128 males and 118 females) and age (**B panel**: 15 juveniles, 33 subadults, and 200 adults). Further detailed information is shown in Figure 1.

Out of 250 pinna samples collected over the past decade, which were all from squirrels from Scotland, the presence of *M. lepromatosis* DNA was detected in 51 (Ct values ≤ 30), accounting for 20.4% of the samples. Additionally, 25 samples (10%) were deemed indeterminate (30 < Ct value < 35), while 174 tested negative (Ct value ≥ 35) (Figure 1A–C). There was no statistically significant difference in the proportion of *M. lepromatosis* presence between male (20/128 = 15.6%) and female (30/118 = 25.4%) squirrels, but the proportion in adults (49/200 = 24.5%) was higher than that in juveniles (2/33 = 6.1%, *p* < 0.05) and subadults (0/15 = 0%, *p* < 0.05) (Figure 2).

**Figure 3:**
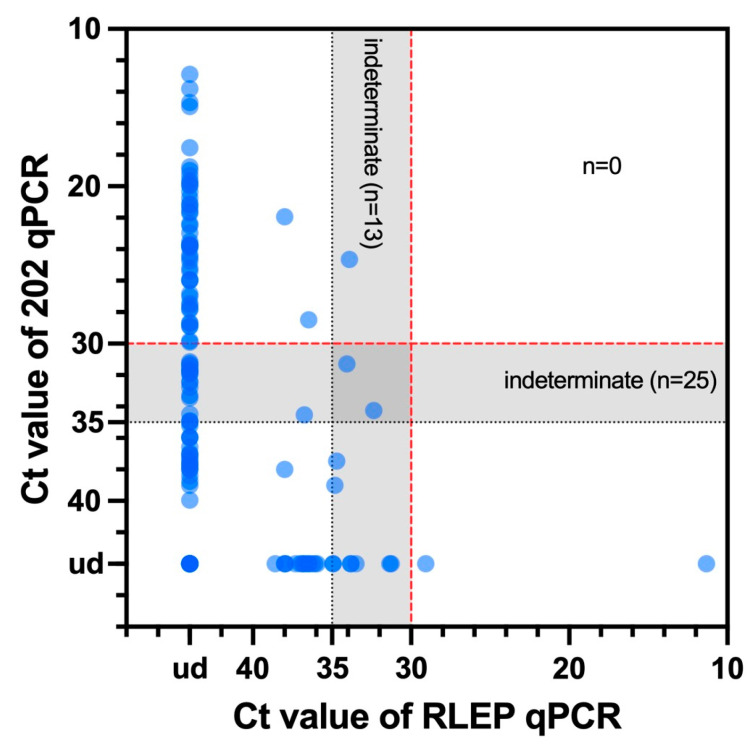
Summary of the RLEP and 202 qPCR results. The Ct values of 202 qPCR for each animal are represented on the *y*-axis, and the Ct values of RLEP qPCR for each animal are represented on the *x*-axis. Further detailed information is shown in Figure 1. Ct values ≤ 30 were considered positive, Ct values ≥ 35 were considered negative, and 30 < Ct values < 35 were considered indeterminate for leprosy bacilli DNA. ud, undetermined.

*M. leprae* DNA was detected in only two pinna samples, both from carcasses found in 2015, accounting for 0.8% of the 250 cases from which pinnae were collected. Thirteen samples were indeterminate, while the remaining 235 tested negative for *M. leprae* DNA (Figure 1D–F). There was no co-detection of both kinds of leprosy bacilli in any squirrels (Figure 3).

Overall, *M. leprae* or *M. lepromatosis* DNA was detected in 21.2% (53/250) of pinna samples from these Scottish red squirrels (excluding the samples categorized as indeterminate). The squirrels all showed post-mortem findings consistent with a cause of death other than leprosy.

### 3.2. Genotyping of M. leprae

To gain a deeper insight into the bacterial origin, we proceeded to ascertain the *M. leprae* genotypes in squirrel samples with the presence of *M. leprae* (*n* = 2) [20,50,51]. One of the samples was determined to be SNP type 3 (Ct value = 11.3), while the range of the other sample was narrowed down to SNP type 3 or 4 due to locus 1 being unidentifiable, likely because of a low amount of DNA (Ct value = 29.1) (Table 1).

### 3.3. Detection of Anti-M. leprae PGL-I Antibodies in the Blood/Body Cavity Fluid

**Figure 4:**
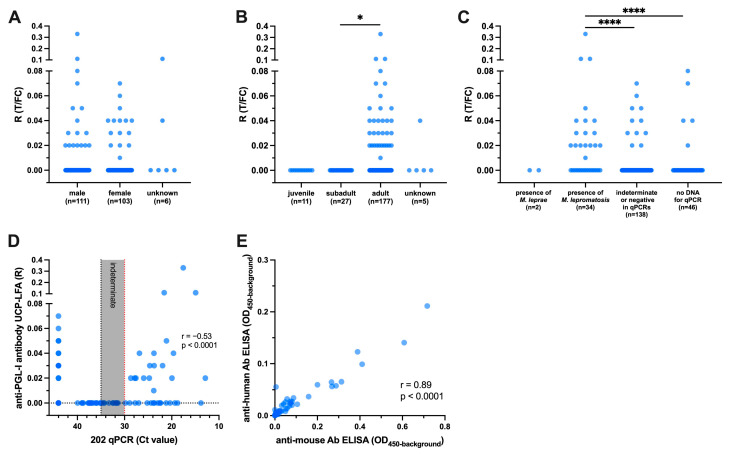
Anti-PGL-I antibody levels in red squirrels. Anti-PGL-I antibody levels were measured by UCP-LFA in blood/body cavity fluid samples from red squirrels (n = 220). UCP-LFA results are displayed as the ratio value (R) between test (T) and flow control (FC) levels. The results were categorized and analyzed based on squirrel sex (**A**), age (**B**), and qPCR outcome (**C**). The correlation of anti-PGL-I antibody UCP-LFA and 202 qPCR is shown in (**D**) (*n* = 174); details of 202 qPCR shown in Figure 1. Fluid samples with ratio values (*n* = 34) in UCP-LFA, positive in RLEP qPCR (*n* = 2), Ct values < 20 in 202 qPCR (n = 3), and negative in two qPCR assays (*n* = 5) were measured by ELISA using anti-human and anti-mouse antibodies (*n* = 44, **E**). The ELISA results of anti-PGL-I antibody are displayed as optical density at 450 nm corrected for background OD values (OD_450-background_). Differences between groups were determined by Kruskal–Wallis test with Dunn’s correction for multiple testing (**A**–**C**); *p*-values: *, *p* < 0.05; ****, and *p* < 0.0001 (**A**). The “r” value is the Spearman correlation coefficient (**D**,**E**).

Next, we assessed the levels of anti-PGL-I antibody in blood/body cavity fluid from 220 red squirrels, comprising 216 from Scotland and four from northern England, using the UCP-LFA developed for the detection of human anti-PGL-I IgM. Detectable anti-PGL-I antibody levels were observed for 34 (15.5%) blood/body cavity fluid samples, including one from a northern English squirrel, albeit mostly at low levels (Figure 4A,B). No difference in antibody levels between male and female squirrels were observed (Figure 4A), while detectable antibody levels were found only in adult squirrels (Figure 4B).

Anti-PGL-I antibodies were not detected in samples from the two animals with Ct ≤ 30 in *M. leprae* qPCR (Ct = 11.3 and 29.1, respectively). In total, 52.9% (18/34) of animals with the presence of *M. lepromatosis* also showed detectable anti-PGL-I antibody levels (Figure 4C). Three squirrels with anti-PGL-I antibody levels R ≥ 0.1 also exhibited high loads of *M. lepromatosis* DNA (Ct values < 21.6) (Figure 4D).

To assess the impact of different species-specific antibodies on detection performance, we selected these 34 samples with detectable antibody levels in the UCP-LFA and an additional 10 samples with no antibody levels as controls, which included those samples positive in RLEP qPCR (*n* = 2), samples with low Ct values (< 20) in 202 qPCR (*n* = 3), and negative samples in two qPCRs (*n* = 5). These samples were then tested using ELISA with either anti-human (as applied in UCP-LFA) or mouse (rodent)-specific antibodies, the latter being more closely related to squirrels. Both ELISAs were able to detect levels of anti-PGL-I antibody, and they exhibited a high correlation (r = 0.89, *p* < 0.0001). Importantly, the values obtained using the anti-mouse antibody were generally higher compared to those using the anti-human antibody (Figure 4E).

### 3.4. Summary of Molecular and Serological Analysis

**Figure 5:**
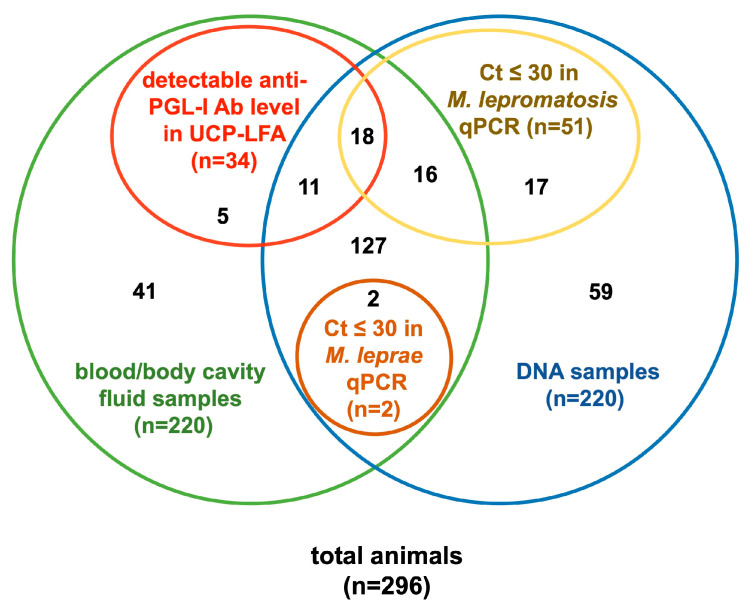
Venn diagram of results from molecular and serological analyses. Details shown in Table 2.

**Figure 6:**
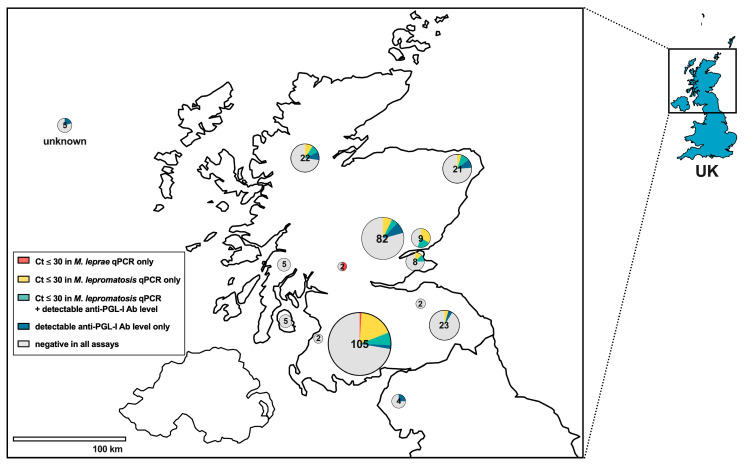
Geographical distribution of leprosy bacillus assay outcomes for red squirrels from the northern UK. Pie charts indicate the regions where squirrels were found and are color-coded for assay outcomes as indicated in the legend box. Numbers within circles indicate the number of animals tested. Squirrels submitted from unknown locations in Scotland are indicated on the upper left. Ab, antibody.

Out of 296 squirrels, 67 (22.6%) had either a Ct ≤ 30 in at least one of the qPCR assays or detectable anti-PGL-I antibody levels in UCP-LFA. Among the animals with both pinna and blood/body cavity fluid (*n* = 174), 18 (10.3%) had Ct ≤ 30 in *M. lepromatosis* qPCR and detectable anti-PGL-I antibody levels in UCP-LFA, but none of these animals showed any signs of leprosy (Table 2, Figure 5). The geographical distribution of all test outcomes shows that the squirrels in which leprosy bacilli were detected were distributed across the northern UK (Figure 6).

## 4. Discussion

In this study, we detected, for the first time, the presence of *M. leprae* DNA in two red squirrels from the Scottish mainland, while previous reports of detection of infected squirrels were limited to the Isle of Arran [40,41]. Additionally, we identified 51 squirrels with *M. lepromatosis* DNA in tissue (pinna) samples, probably consistent with asymptomatic infection, given that leprosy was not suspected at post-mortem examination of these animals, and they were considered to have died from other causes. Overall, leprosy bacilli were detected in pinna samples from 21.2% (53 out of 250) of the red squirrel samples tested. These findings are in line with a study conducted in 2016 [41], reporting leprosy bacilli in 21% of 101 squirrels without clinical signs, from around the British Isles. Considering the relatively short average lifespan of wild red squirrels of approximately 4–5 years [52], it can be inferred that the squirrels were infected within recent years. This indicates that leprosy bacilli remain present amongst the Scottish and northern English red squirrel population.

*M. leprae* displays some cross-species transmission, affecting non-human primates (NHP) [53,54] and armadillos [55,56,57] through direct contact with humans. Individuals who rely on armadillos as a protein source may also acquire *M. leprae* infection through the food chain, particularly when meat is consumed raw or when food preparation includes contact with infected blood [57]. Despite human leprosy cases having disappeared in the UK for centuries, it is clear that leprosy bacilli are continuing to persist here long-term, with a potential risk to public health. The fact that more than one-fifth of this sampled red squirrel population demonstrated evidence of infection suggests that the species may serve as a maintenance host for leprosy bacilli. Alternatively, it is possible that red squirrels are relatively frequently being exposed to infection from another animal or environmental reservoir. The ecology of disease transmission merits further investigation, in light of its zoonotic potential and because of the red squirrel’s unfavorable conservation status in the UK.

It should be noted that there are no known reports of zoonotic transmission of leprosy bacilli from squirrels to humans, and in both humans and squirrels, the potential transmission routes (anthroponotic or zoonotic) of leprosy bacilli remain unknown [58]. *M. leprae* strains previously identified in red squirrels on Brownsea Island are closely related to those that caused leprosy in medieval Europeans [41,59], with one strain traced back to the skeletal remains of a leprosy patient from around 730 years ago in Winchester, England [41]. Further historical links between *M. leprae* strains (both SNP subtype 3I) in red squirrels and humans have also recently been made through examination of historical squirrel remains from Winchester [58]. In our study, two cases of *M. leprae* were identified as SNP type 3 and type 3 or 4, respectively, through genotyping, consistent with previous findings. Further genome sequencing could enhance our understanding of the genetic diversity and historical context of these strains in red squirrels and their role in transmission.

*M. lepromatosis*, on the other hand, is a recently discovered pathogen, and our knowledge of its cross-species transmission is limited. Phylogenetic comparisons indicated that *M. lepromatosis* strains from Scotland and Mexico diverged from a common ancestor around 27,000 years ago [41]. In our study, *M. lepromatosis* was found in red squirrels despite no documented human infections in the UK. Moreover, in previous studies in areas of the UK where red squirrel reside, *M. lepromatosis* was not detected in other wild rodents [60] or in the soil [41,51]. Additionally, no wild armadillos in regions where human infections with *M. lepromatosis* have occurred have been found to have this bacillus [8,44]. Ongoing monitoring and further research in wildlife (not limited to squirrels) is necessary to investigate the routes of transmission and environmental reservoirs facilitating persistence of these mycobacteria [44].

The impact of infection with leprosy bacilli on red squirrel populations is currently unknown. Previous observations indicated that leprosy is a relatively chronic, protracted disease as opposed to an acute cause of mortality [26]. However, a greater understanding of leprosy bacilli infection is important for assessing potential risks to red squirrel populations and for informing conservation efforts.

The method we developed to measure the levels of anti-PGL-I antibody by UCP-LFA for detecting *M. leprae* infection has previously been successfully applied to longitudinal monitoring of infection with leprosy bacilli in red squirrels, showing that antibody levels corresponded to the development of clinical signs [26,27]. However, we also observed that anti-PGL-I antibody levels in post-mortem blood/body cavity fluids (even when immediately frozen) were substantially lower than those in blood or serum from living squirrels [27]. The samples used for this study were all collected from dead animals and showed overall lower anti-PGL-I antibody levels compared to samples derived from living squirrels [26,27]. Additionally, similar to findings in children for whom antibody levels and seropositivity percentage increase with age [49], only the carcasses of adult squirrels in our study showed detectable anti-PGL-I antibody levels by UCP-LFA, whereas no antibody levels were detected in subadult squirrels, although a few (*n* = 2) subadults had the presence of leprosy bacilli DNA. However, the kinetics of anti-PGL-I serology in squirrels after infection with leprosy bacteria remain unclear, highlighting the importance of using molecular techniques to directly detect the pathogen in addition to serology for disease surveillance in these animals.

Comparing the detection of anti-PGL-I antibodies in red squirrels using anti-human with anti-mouse antibodies showed high correlation, although the anti-mouse antibody yielded higher levels. This was likely due to the higher affinity of the anti-mouse antibody for squirrel anti-PGL-I antibodies. Thus, to obtain higher sensitivity for squirrel screening, anti-mouse antibodies could be applied to the UCP-LFA platform in the future to detect anti-PGL-I antibodies in red squirrels.

Molecular diagnostics, such as the multiplex qPCR used in this study, provide direct evidence of pathogen presence, can distinguish between pathogen strains, and can be combined with genotyping and genome sequencing to elucidate pathogen transmission routes [41,50,61,62]. They are not limited by host species [54,63,64], and, relative to serological tests, are less affected by the age of the samples, with detections possible even in ancient skeletons of humans as well as squirrels [58,62]. However, given the obligate intracellular nature of infection with leprosy bacilli, molecular tests typically require the collection of tissue samples; thus, they are less suitable for dynamic monitoring of live squirrels but are rather suitable for post-mortem sampling. Furthermore, positive qPCR results are also restricted by bacterial load in (heterogenous) sampled tissues. Serological testing using the UCP-LFA platform, on the other hand, detects specific antibodies directed against leprosy bacilli, requiring (in a live animal) only a minimal amount of blood sample, and, as such, is relatively less invasive. Serology can be used for longitudinal monitoring in field research [23,24,27,47,48]. However, because both *M. leprae* and *M. lepromatosis* produce anti-PGL-I antibodies [19], antibody detection cannot distinguish between infection with either of these two species. Furthermore, although more important in humans than in squirrels, anti-PGL-I serology cannot distinguish between past and present infection. Depending on the experimental purpose and sample type, choosing the appropriate testing method, or combining both, can enhance the performance of detection of these mycobacterial infections [26].

## 5. Conclusions

This study investigated carcasses of Eurasian red squirrels for the presence of *M. leprae* and *M. lepromatosis*, using molecular methods including 202 qPCR and RLEP qPCR, as well as serological testing for anti-PGL-I antibodies by UCP-LFA. The results obtained from all methods indicated that 22.6% of these carcasses were qPCR positive, whereas 15.5% showed ant-PGL-I antibodies, suggesting the ongoing presence of leprosy bacilli in red squirrels in Scotland and northern England. Therefore, continued monitoring using both molecular and serological approaches is recommended to further research the ecology of leprosy in red squirrels and other potential wildlife hosts, to gain insight into the potential for the zoonotic transmission of leprosy bacilli, and to inform red squirrel conservation efforts.

## Figures and Tables

**Table 1:** SNP typing results.

Code	Locus 1	Locus 2	Locus 3	Genotype
R25/15	C	T	C	3
R26/15	ud	T	C	3 or 4

Polymorphic sites in the genome of *M. leprae*: locus 1 (SNP-14676), locus 2 (SNP-1642875), locus 3 (SNP-2935685), and the corresponding genotype. Nucleic acid (T, thymine; C, cytosine) corresponding to each polymorphic site of *M. leprae* from DNA derived from red squirrels were sequenced. When PCR amplification or sequencing of the locus was not successful it was marked as undetermined (ud).

**Table 2:** Summary of molecular and serological analysis.

RLEP qPCR (*M. leprae*)	202 qPCR (*M. lepromatosis*)	UCP-LFA (Anti-PGL-I ANTIBODIES)	Squirrels (n)
+	−	−	2
−	+	+	18
−	+	−	16
−	−	+	11
−	−	−	127
−	+	n.a.	17
−	−	n.a.	59
n.a.	n.a.	+	5
n.a.	n.a.	−	41

The results of molecular and serological analysis were summarized. “+” and “−” in qPCRs indicates animals with Ct value ≤ 30 and >30, respectively; “+” and “−” in UCP-LFA indicates animals with detectable and undetectable anti-PGL-I antibody levels, respectively. The results are visualized in a Venn diagram shown in Figure 5. More detailed descriptions can be found in Figure 1 and Figure 4. Ab, antibodies; n.a., not applicable.

## Data Availability

Data are contained within the article and Supplementary Materials.

## Supplementary Materials

The following supporting information can be downloaded at: https://www.mdpi.com/article/10.3390/ani14132005/s1; Table S1: Characteristics of red squirrels; Table S2: Primer and probe sequences for RLEP and 202 TaqMan qPCR assays.
