# Supplementary material for: Molecular and Serological Surveillance for Mycobacterium leprae and Mycobacterium lepromatosis in Wild Red Squirrels (Sciurus vulgaris) from Scotland and Northern England
Source: Animals (Basel). 2024 Jul 7;14(13):2005. doi: 10.3390/ani14132005 (PMC11240566; doi:10.3390/ani14132005)
Supplement: Supplementary file 1 [file animals-14-02005-s001.zip › animals-3067122-supplementary.pdf]

Molecular and Serological Surveillance for *Mycobacterium leprae* and *Mycobacterium lepromatosis* in Wild Red Squirrels (*Sciurus vulgaris*) from Scotland and Northern Engalnd

Zijie Zhou, Anouk van Hooij, Gaby N. Wassenaar, Emma Seed, Els M. Verhard-Seymonsbergen, Paul L.A.M. Corstjens, Anna L. Meredith, Liam A. Wilson, Elspeth M. Milne, Katie M. Beckmann, and Annemieke Geluk

Supplementary Table S1. *Characteristics of red squirrels*

Squirrel Ref.	Year of post-mortem	Year found	Sex	Age	Blood/body cavity fluid samples (1=yes)	Pinna (1=yes)	Animals have both blood/ body cavity fluid and pinna samples (1=yes)
1/2004	2004	-	-	-	1		
1/2006	2006	-	-	-	1		
1/2007	2007	-	-	-	1		
1/2010	2010	-	-	-	1		
R42/14	2014	2014	M	Adult	1		
R43/14	2014	2014	F	Subadult	1		
R44/14	2014	2014	M	Adult	1		
R45/14	2014	2014	M	Adult	1		
R46/14	2014	2014	F	Adult		1	
R47/14	2014	2014	F	Subadult		1	
R48/14	2014	2014	F	Adult		1	
R2/15	2015	2014	F	Subadult	1	1	1
R3/15	2015	2014	F	<16 weeks	1	1	1
R5/15	2015	2014	M	<16 weeks	1		
R7/15	2015	2015	M	Adult		1	
R8/15	2015	2015	M	Adult		1	
R10/15	2015	2015	M	Adult		1	
R12/15	2015	2015	M	Adult		1	
R13/15	2015	2015	F	Adult		1	
R14/15	2015	2015	M	Subadult		1	
R15/15	2015	2015	M	Adult		1	
R16/15	2015	2015	M	Adult		1	
R17/15	2015	2015	M	Adult		1	
R18/15	2015	2015	F	Adult		1	
R21/15	2015	2015	M	<16 weeks	1	1	1
R22/15	2015	2015	M	Adult	1	1	1
R23/15	2015	2015	M	Adult		1	
R24/15	2015	2015	M	Adult	1	1	1
R25/15	2015	2015	F	Adult	1	1	1
R26/15	2015	2015	M	Adult	1	1	1
R27/15	2015	2015	M	Subadult		1	
R28/15	2015	2015	F	Adult	1	1	1
R29/15	2015	2015	M	Adult	1	1	1
R30/15	2015	2015	F	Adult	1	1	1
R31/15	2015	2015	F	Adult	1	1	1
R19/16	2016	2016	M	-	1	1	1
R20/16	2016	2016	F	Adult	1	1	1
R21/16	2016	2016	F	Adult	1	1	1

Squirrel Ref.	Year of post-mortem	Year found	Sex	Age	Blood/body cavity fluid samples (1=yes)	Pinna (1=yes)	Animals have both blood/ body cavity fluid and pinna samples (1=yes)
R22/16	2016	2014	M	Adult	1	1	1
R23/16	2016	2015	F	Adult	1	1	1
R24/16	2016	2013	M	Adult	1	1	1
R25/16	2016	2015	F	Adult	1	1	1
R26/16	2016	2016	F	Adult	1	1	1
R27/16	2016	2016	F	Subadult	1	1	1
R28/16	2016	2016	F	Adult		1	
R29/16	2016	2016	F	Adult	1		
R2/17	2017	2016	F	<16 weeks	1		
R4/17	2017	2017	F	Adult	1		
R5/17	2017	2017	M	Adult	1		
R9/17	2017	2017	F	Adult	1		
R11/17	2017	2017	F	Adult		1	
R12/17	2017	2017	M	Adult	1	1	1
R13/17	2017	2017	M	Subadult	1		
R14/17	2017	2017	F	Adult	1		
R17/17	2017	2017	F	Adult	1	1	1
R18/17	2017	2017	F	Adult		1	
R19/17	2017	2017	F	Adult		1	
R20/17	2017	2017	F	Adult	1		
R1/18	2018	2018	M	Adult	1	1	1
R2/18	2018	2018	F	Adult	1	1	1
R3/18	2018	2018	M	Adult	1	1	1
R4/18	2018	2018	M	Adult	1	1	1
R5/18	2018	2018	M	Adult	1		
R6/18	2018	2018	F	Adult	1		
R7/18	2018	2018	M	Adult	1		
R9/18	2018	2014	M	Subadult	1		
R11/18	2018	2018	F	Adult	1		
R12/18	2018	2018	M	Adult	1		
R13/18	2018	2018	M	Adult	1	1	1
R14/18	2018	2018	M	Adult	1	1	1
R15/18	2018	2018	F	Subadult	1	1	1
R17/18	2018	2018	F	Adult	1	1	1
R19/18	2018	2018	M	Adult	1	1	1
R20/18	2018	2018	M	Adult	1	1	1
R21/18	2018	2018	M	Subadult	1	1	1
R22/18	2018	2018	F	Adult	1	1	1
R24/18	2018	2018	M	Adult	1		

Squirrel Ref.	Year of post-mortem	Year found	Sex	Age	Blood/body cavity fluid samples (1=yes)	Pinna (1=yes)	Animals have both blood/ body cavity fluid and pinna samples (1=yes)
R25/18	2018	2018	M	<16 weeks	1		
R26/18	2018	2018	M	Adult		1	
R27/18	2018	2018	F	Adult	1		
R28/18	2018	2018	F	Adult	1		
R29/18	2018	2018	F	Adult	1	1	1
R30/18	2018	2018	F	Adult	1	1	1
R31/18	2018	2018	F	Adult	1		
R32/18	2018	2018	F	Subadult	1		
R33/18	2018	2018	M	Adult		1	
R35/18	2018	2018	F	Adult	1	1	1
R36/18	2018	2018	M	Adult	1	1	1
R40/18	2018	2018	F	Subadult	1	1	1
R41/18	2018	2018	M	Adult		1	
R42/18	2018	2018	M	Subadult	1		
R43/18	2018	2018	M	Adult	1		
R44/18	2018	2018	M	Adult	1		
R1/19	2019	2019	F	Subadult	1		
R2/19	2019	2019	M	Adult	1		
R3/19	2019	2018	M	Adult	1		
R4/19	2019	2017	F	Adult	1		
R5/19	2019	2018	F	Adult	1		
R7/19	2019	2019	M	Adult	1		
R8/19	2019	2019	M	Adult	1		
R14/19	2019	2019	F	Subadult	1		
R15/19	2019	2019	M	Adult	1		
R17/19	2019	2019	F	Adult	1		
R21/19	2019	2019	M	Adult	1	1	1
R24/19	2019	2019	M	Adult		1	
R26/19	2019	2019	M	Adult		1	
R27/19	2019	2019	F	Subadult		1	
R28/19	2019	2019	M	Subadult	1	1	1
R29/19	2019	2019	M	<16 weeks		1	
R30/19	2019	2019	M	Subadult	1	1	1
R31/19	2019	2019	F	<16 weeks		1	
R32/19	2019	2019	M	Adult	1	1	1
R33/19	2019	2019	M	Adult		1	
R34/19	2019	2019	M	Adult		1	
R35/19	2019	2019	F	Adult	1	1	1
R36/19	2019	2019	F	Adult		1	

Squirrel Ref.	Year of post-mortem	Year found	Sex	Age	Blood/body cavity fluid samples (1=yes)	Pinna (1=yes)	Animals have both blood/ body cavity fluid and pinna samples (1=yes)
R37/19	2019	2019	F	Adult		1	
R38/19	2019	2019	M	Adult	1	1	1
R39/19	2019	2019	M	<16 weeks	1	1	1
R40/19	2019	2019	M	Adult	1	1	1
R41/19	2019	2019	F	Subadult	1	1	1
R42/19	2019	2019	F	Adult	1	1	1
R43/19	2019	2019	M	Adult	1	1	1
R44/19	2019	2019	F	Adult	1	1	1
R45/19	2019	2019	M	Subadult	1	1	1
R46/19	2019	2019	M	<16 weeks		1	
R47/19	2019	2019	F	<16 weeks	1	1	1
R48/19	2019	2019	F	Adult	1	1	1
R49/19	2019	2019	F	<16 weeks		1	
R50/19	2019	2019	F	Adult		1	
R51/19	2019	2019	F	Adult		1	
R52/19	2019	2019	F	Adult		1	
R53/19	2019	2019	F	Adult		1	
R54/19	2019	2019	M	Adult	1	1	1
R55/19	2019	2019	F	Adult		1	
R56/19	2019	2019	M	<16 weeks		1	
R57/19	2019	2019	M	Adult		1	
R58/19	2019	2019	F	Adult	1	1	1
R59/19	2019	2019	M	Adult	1	1	1
R60/19	2019	2019	M	Adult		1	
R1/20	2020	2020	F	Adult	1	1	1
R2/20	2020	2019	F	Adult		1	
R3/20	2020	2020	F	Adult	1	1	1
R4/20	2020	2020	M	Adult	1	1	1
R5/20	2020	2020	M	Adult	1	1	1
R6/20	2020	2020	F	Adult	1	1	1
R7/20	2020	2020	F	Adult	1	1	1
R8/20	2020	2020	M	Adult		1	
R9/20	2020	2020	F	Subadult	1	1	1
R10/20	2020	2020	M	Subadult		1	
R11/20	2020	2020	M	Adult		1	
R12/20	2020	2020	M	Adult	1	1	1
R13/20	2020	2020	M	Adult	1	1	1
R14/20	2020	2020	F	Adult	1	1	1
R15/20	2020	2020	M	Adult	1	1	1

Squirrel Ref.	Year of post-mortem	Year found	Sex	Age	Blood/body cavity fluid samples (1=yes)	Pinna (1=yes)	Animals have both blood/ body cavity fluid and pinna samples (1=yes)
R16/20	2020	2020	F	Adult	1	1	1
R17/20	2020	2020	M	Subadult		1	
R18/20	2020	2020	F	Subadult		1	
R19/20	2020	2020	F	Adult	1	1	1
R20/20	2020	2020	M	Subadult	1	1	1
R21/20	2020	2020	M	Adult	1	1	1
R22/20	2020	2020	F	Adult	1	1	1
R23/20	2020	2020	F	Adult	1	1	1
R24/20	2020	2020	M	Adult	1	1	1
R25/20	2020	2020	F	Adult	1	1	1
R26/20	2020	2020	M	Subadult		1	
R27/20	2020	2020	F	Adult		1	
R28/20	2020	2020	F	Adult	1	1	1
R29/20	2020	2020	M	Adult	1	1	1
R30/20	2020	2020	F	Adult	1	1	1
R31/20	2020	2020	M	Subadult	1	1	1
R32/20	2020	2020	F	Adult		1	
R33/20	2020	2020	M	Adult	1	1	1
R34/20	2020	2020	F	Adult	1	1	1
R35/20	2020	2020	M	Adult		1	
R36/20	2020	2020	M	Adult		1	
R37/20	2020	**2020**	M	Subadult		1	
R38/20	2020	2020	M	Adult		1	
R39/20	2020	2020	F	Adult		1	
R40/20	2020	2020	M	Adult		1	
R41/20	2020	2020	M	Adult		1	
R42/20	2020	2020	M	Adult	1	1	1
R43/20	2020	2020	F	Adult		1	
R44/20	2020	2020	F	Adult	1	1	1
R45/20	2020	2020	M	Adult	1	1	1
R46/20	2020	2020	M	Adult	1	1	1
R47/20	2020	2020	F	Subadult	1	1	1
R48/20	2020	2020	M	Adult	1	1	1
R1/21	2021	2020	F	Adult		1	
R2/21	2021	2021	F	Adult		1	
R3/21	2021	2021	F	Adult	1	1	1
R4/21	2021	2020	F	Subadult	1	1	1
R5/21	2021	2021	M	Adult	1	1	1
R6/21	2021	2021	M	Adult	1	1	1

Squirrel Ref.	Year of post-mortem	Year found	Sex	Age	Blood/body cavity fluid samples (1=yes)	Pinna (1=yes)	Animals have both blood/ body cavity fluid and pinna samples (1=yes)
R7/21	2021	2020	M	Adult	1	1	1
R8/21	2021	2021	M	Adult	1	1	1
R9/21	2021	2021	F	Adult		1	
R11/21	2021	2021	M	Adult	1	1	1
R12/21	2021	2021	F	Adult	1	1	1
R13/21	2021	2021	M	Adult	1	1	1
R14/21	2021	2021	F	Adult	1	1	1
R15/21	2021	2021	M	Adult	1	1	1
R16/21	2021	2021	M	Adult	1	1	1
R18/21	2021	2021	-	Adult	1	1	1
R19/21	2021	2021	F	Adult	1	1	1
R20/21	2021	2021	F	Adult	1		
R21/21	2021	2021	F	Adult	1	1	1
R22/21	2021	2020	M	Adult	1	1	1
R23/21	2021	2021	M	Adult	1	1	1
R24/21	2021	2021	F	Adult	1	1	1
R25/21	2021	2021	M	Adult		1	
R26/21	2021	2021	F	Subadult		1	
R27/21	2021	2021	F	Adult	1		
R28/21	2021	2021	F	Adult	1	1	1
R29/21	2021	2021	M	Adult	1	1	1
R30/21	2021	2021	-	Adult	1	1	1
R31/21	2021	2021	M	Adult	1	1	1
R32/21	2021	2021	M	Adult	1	1	1
R33/21	2021	2021	-	Adult		1	
R34/21	2021	2021	M	Adult		1	
R35/21	2021	2021	M	Adult	1	1	1
R36/21	2021	2021	M	Adult	1		
R37/21	2021	2021	M	Adult	1	1	1
R38/21	2021	2021	M	Subadult	1	1	1
R39/21	2021	2021	F	Adult	1	1	1
R40/21	2021	2021	M	Adult	1	1	1
R41/21	2021	2021	M	Adult	1	1	1
R42/21	2021	2021	F	Adult	1	1	1
R43/21	2021	2021	M	Adult	1	1	1
R44/21	2021	2021	M	Subadult	1	1	1
R45/21	2021	2021	M	Adult	1	1	1
R46/21	2021	2021	F	<16 weeks		1	
R47/21	2021	2021	M	Adult	1		

Squirrel Ref.	Year of post-mortem	Year found	Sex	Age	Blood/body cavity fluid samples (1=yes)	Pinna (1=yes)	Animals have both blood/ body cavity fluid and pinna samples (1=yes)
R48/21	2021	2021	M	Adult	1	1	1
R49/21	2021	2021	M	Adult	1	1	1
R50/21	2021	2021	M	Adult	1	1	1
R51/21	2021	2021	F	Adult	1	1	1
R52/21	2021	2021	F	<16 weeks		1	
R53/21	2021	2021	M	Subadult	1	1	1
R55/21	2021	2021	-	-		1	
R1/22	2022	2022	M	Subadult		1	
R2/22	2022	2022	M	Adult	1	1	1
R3/22	2022	2022	F	Adult		1	
R4/22	2022	2022	F	Adult	1	1	1
R5/22	2022	2021	M	Adult	1	1	1
R6/22	2022	2022	F	Adult	1	1	1
R7/22	2022	2021	M	Adult	1	1	1
R8/22	2022	2022	F	Subadult	1	1	1
R9/22	2022	2022	M	Adult	1	1	1
R10/22	2022	2013	M	Adult		1	
R11/22	2022	2015	F	Adult	1	1	1
R12/22	2022	2015	F	Adult	1	1	1
R13/22	2022	2016	M	Adult	1	1	1
R14/22	2022	2016	F	Adult	1	1	1
R15/22	2022	2016	F	Adult	1	1	1
R16/22	2022	2017	F	Adult	1	1	1
R17/22	2022	2017	F	Adult	1	1	1
R18/22	2022	2018	M	Adult	1	1	1
R19/22	2022	2017	F	Adult	1	1	1
R20/22	2022	2019	M	Adult	1	1	1
R21/22	2022	2020	M	Adult	1	1	1
R22/22	2022	2021	M	Adult	1	1	1
R23/22	2022	2018	M	Adult	1	1	1
R24/22	2022	2022	M	Subadult		1	
R25/22	2022	2022	F	Adult	1	1	1
R26/22	2022	2022	F	Subadult		1	
R27/22	2022	2022	F	Adult	1	1	1
R28/22	2022	2022	F	Adult	1	1	1
R29/22	2022	2022	F	Adult	1	1	1
R30/22	2022	2022	F	Adult	1	1	1
R31/22	2022	2022	M	Adult	1	1	1
R32/22	2022	2022	M	<16 weeks	1	1	1

Squirrel Ref.	Year of post-mortem	Year found	Sex	Age	Blood/body cavity fluid samples (1=yes)	Pinna (1=yes)	Animals have both blood/ body cavity fluid and pinna samples (1=yes)
R33/22	2022	2022	M	Subadult	1	1	1
R34/22	2022	2022	M	<16 weeks	1	1	1
R35/22	2022	2022	F	Subadult	1	1	1
R36/22	2022	2022	F	Subadult		1	
R38/22	2022	2022	F	Adult	1	1	1
R39/22	2022	2022	F	Adult	1	1	1
R40/22	2022	2022	F	Adult	1	1	1
R41/22	2022	2022	F	<16 weeks	1	1	1
R42/22	2022	2022	F	<16 weeks	1	1	1
R1/23	2023	2023	F	Adult		1	
R2/23	2023	2023	M	Adult	1	1	1
R3/23	2023	2023	M	Adult		1	
R4/23	2023	2023	F	Adult		1	
R5/23	2023	2023	F	Adult	1	1	1
R6/23	2023	2023	F	Adult	1	1	1
R7/23	2023	2023	F	Adult	1	1	1
R8/23	2023	2023	F	Adult	1	1	1
R9/23	2023	2023	F	Adult	1	1	1
R10/23	2023	2023	M	Adult	1	1	1
R11/23	2023	2023	M	Adult	1	1	1
R12/23	2023	2023	M	Adult	1	1	1
R13/23	2023	2023	F	Adult	1	1	1
R14/23	2023	2023	M	Adult	1	1	1
R15/23	2023	2022	M	Adult	1	1	1
R16/23	2023	2023	F	Adult	1	1	1
**TOTAL**					**220**	**251**	**174**

M, male; F, female; -, information unknown.

Assessment of the age of the squirrels was based on a previously reported method [1]. Each squirrel was classified as adult, subadult or juvenile on the basis of its bodyweight, crown-rump length and the degree of maturity of the hair coat and reproductive organs.

Supplementary Table S2. *Primer and probe sequences for RLEP and 202 TaqMan qPCR assays*

Target	Primer/ Probe	Sequence
RLEP	RLEP qPCR F	5’- GCAGCAGTATCGTGTTAGTGAA -3’
	RLEP qPCR R	5’- CGCTAGAAGGTTGCCGTAT -3’
	RLEP qPCR probe FAM	5’- FAM-CGCCGACGGCCGGATCATCGA -3’
202	202 qPCR F	5’- CTGATCGCACACCTTGATGAGAG -3’
	202 qPCR R	5’- GTTAGGTTGATCGACATCTTCGGTGC -3’
	202 qPCR probe VIC	5’- VIC- CACCACTAGCGCACCACGTCAGACAGGC -3’

References:

1. Carroll, B.; Russell, P.; Gurnell, J.; Nettleton, P.; Sainsbury, A.W. Epidemics of squirrelpox virus disease in red squirrels (Sciurus vulgaris): temporal and serological findings. *Epidemiol Infect* **2009**, *137*, 257-265, doi:10.1017/s0950268808000836.
